# Gastric Juice Expression of Th-17 and T-Reg Related Cytokines in Scleroderma Esophageal Involvement

**DOI:** 10.3390/cells9092106

**Published:** 2020-09-16

**Authors:** Stefania Nicola, Giovanni Rolla, Caterina Bucca, Giada Geronazzo, Irene Ridolfi, Andrea Ferraris, Enrico Fusaro, Clara Lisa Peroni, Luca Dughera, Luisa Brussino

**Affiliations:** 1 Department of Medical Sciences, Allergy and Clinical Immunology Unit, University of Torino & Mauriziano Hospital, 10128 Turin, Italy; stefania.nicola@edu.unito.it (S.N.); grolla@mauriziano.it (G.R.); caterina.bucca@unito.it (C.B.); giada.geronazzo@edu.unito.it (G.G.); irene.ridolfi@edu.unito.it (I.R.); 2 Division of Diagnostic Imaging, Department of Surgical Sciences, Città della Salute e della Scienza Hospital, University of Turin, 10126 Turin, Italy; andrea.ferraris@alice.it; 3 Rheumatology Department, Azienda Ospedaliera Città della Salute e della Scienza di Torino, 10126 Turin, Italy; efusaro@cittadellasalute.to.it (E.F.); cperoni@cittadellasalute.to.it (C.L.P.); 4 Unit of Digestive Motility and Endoscopy, Department of Medicine, Città della Salute e della Scienza, 10126 Turin, Italy; luca.dughera@libero.it

**Keywords:** systemic sclerosis, T-reg, Th-17, endothelin-1, esophagus

## Abstract

Background: Systemic sclerosis (SSc) is a connective tissue disorder which key feature is a fibrotic process. The role of Endothelin-1 (ET-1) and T-helper (Th)-1 cells in lung and skin fibrosis is well known, although Th17- and Treg-cells were found to be involved. However, no studies analyzed cytokines expression in gastric-juice of SSc patients. Our study aimed to evaluate proinflammatory and profibrotic cytokines in gastric-juice of SSc patients and to investigate their correlations with esophageal dysmotility. Methods: Patients performed upper-gastrointestinal-endoscopy with gastric-juice collection, esophageal manometry and thoracic CT-scan. GM-CSF, ET-1, Th-1 (IFN-γ, IL-1β, TNF-α, IL-2, IL-6, IL-9), Th-17 (IL-17, IL-21, IL-22, IL-23) and T-reg (IL-10, TGF-β) related cytokines were measured in 29 SSc-patients and 20 healthy-controls. Results: Patients showed significant lower levels of IL-6, IL-17, IL-22 and ET-1 (*p* < 0.005) compared with controls. Patients with atrophic gastritis presented significant lower levels of IL-2, IL-9, IL-6, TGF-β, GM-CSF, IL-17 and ET-1 (*p* < 0.005) compared to patients without gastritis. Increased values of IL-2, IL-9, IL-1β, IL-17, ET-1 and GM-CSF (*p* < 0.005) were observed in patients with esophageal impairment. This is the first report of cytokines measurement in gastric juice of patients with SSc. The high IL-17 concentrations in gastric-juice of scleroderma patients with esophageal dysmotility support the signature of Th-17 cells in scleroderma esophageal fibrosis.

## 1. Introduction

Systemic sclerosis (SSc) is a chronic connective tissue disorder, in which humoral and cellular immunity alterations lead to massive fibrosis of the skin and internal organs [1,2].

Gastrointestinal involvement in systemic sclerosis is very common, affecting up to 87% of patients [3], with the esophagus being the most commonly involved tract, followed by rectum and small bowel [4].

Many studies have reported increased levels of profibrotic T-helper (Th)-17 cells and Th-17 related cytokines in serum and skin [5,6] of SSc patients. These cytokines act as profibrotic molecules that promote transforming growth factor TGF-β release, fibroblasts activation, and myofibroblasts stimulation [7], leading to connective tissues remodeling [8].

More recently, several authors also reported decreased frequency and impaired function of circulating CD4^+^, CD25^+^, FoxP3^+^ human regulatory T cells (Tregs) in SSc patients compared to healthy controls and other autoimmune diseases [9,10,11], thus postulating a Treg/Th17 imbalance as the main component of SSc pathogenesis [12].

High serum levels of Th-17 related cytokines were also found to be associated with skin involvement SSc patients with digital ulcers [13]. This led many authors to think that Th-17 cells and Th17-related cytokines might play a key role in promoting lung and skin fibrosis, which are commonly observed in SSc patients [13,14,15,16,17].

Gastric biopsies obtained in SSc patients showed an increased number of CD4^+^ T cells, mast cells, and eosinophils in gastric mucosa [14]. In addition, overexpression of Endothelin (ET)-1 in epithelial cells and vascular walls of scleroderma patients have been associated with the fibrotic changes observed in gastric mucosa [15].

However, whether the concentration of proinflammatory and profibrotic cytokines is increased in gastric juice of patients affected with SSc has not yet been investigated.

In the present study, we measured the concentration of profibrotic molecules (GM-CSF, ET-1), Th-1 (IFN-gamma, IL-1β, TNF-α, IL-2, IL-6, IL-9), Th-17 (IL-17, IL-21, IL-22, IL-23), and T-reg (IL-10, TGF-β) related cytokines in gastric juice of patients affected with SSc, at the aim to investigate the correlation between their levels and esophageal dysfunction.

## 2. Methods

### 2.1. Patients

All patients who met the 2013 ACR/EULAR classification criteria for SSc [18] attending the Outpatients Clinic of Rheumatic Disease between May 2014 and March 2015, were enrolled in the study.

Patients presenting a history of smoking, COPD, asthma, malignancy, or autoimmune disease different from SSc were excluded from the study. In addition, treatment with systemic steroids or immunosuppressive drugs at the screening visit was an exclusion criterion.

All patients underwent esophageal manometry and a thoracic CT scan. Gastric juice collection, endoscopy, and esophageal manometry were performed in the same week for each patient.

Among patients who performed gastroscopy due to epigastric discomfort, twenty people with no abnormalities were recruited as a control group for gastric juice cytokines levels.

Patients and control subjects released their informed consent, and the study was approved by the Institutional Review Board for human studies of our hospital (Comitato Etico Interaziendale Città della Salute e della Scienza di Torino; approval number 0039653, September 2013) in respect of the Helsinki Declaration.

### 2.2. Cytokine Assays

The following cytokines have been measured in gastric juice using a multiplex immunoassay (Bio-Rad Laboratories Inc., Hercules, CA, USA), with Bioplex 100 xMAP technology (Luminex Corp, Austin, TX, USA): GM-CSF, ET-1, IFN-γ, IL-1β, TNF-α, IL-2, IL-6, IL-9, IL-17, IL-21, IL-22, IL-23, IL-10, and TGF-β. Reliability of cytokines measurement at low pH was performed according to another previously published paper [19].

Data analysis was performed using the Bioplex Manager 4.1 software (Bio-Rad Laboratories, Segrate, Italy). All samples were analyzed at the same time, and every 96-well plate included an 8-point standard curve. Cytokines concentrations below the detection limit or outside the linear part of the curve were excluded from the analysis [20].

### 2.3. Upper Gastrointestinal (GI) Endoscopy

All patients and healthy controls underwent upper gastrointestinal endoscopy (Olympus Video Gastroscope 9.2 mm, Waltham, MA, USA) with biopsies. Gastric biopsies were evaluated according to the Operative Link for Gastritis Assessment (OLGA) planning [21] and *Helicobacter pylori* screening was performed in all samples. Gastric juice was collected in two sterile samples and stored at −80 °C until the multiplex analysis.

### 2.4. Esophageal Manometry

Esophageal manometry was performed in all fasting patients using a Given Imaging™ (Yorknimu, Israel) solid-state high-resolution manometry (HRM) system. The HRM catheter was inserted into the nostril and moved up to the stomach. All patients were placed in a supine position and they were asked to swallow 5 mL of water for ten consecutive times. Then, the patients were asked not to swallow for at least 30 s, allowing the signal to be recorded.

Manometric data were analyzed using the ManoView Analysis software (version 3.0; Given Imaging™). Esophageal motility was assessed by recording esophageal pressure parameters according to the Chicago criteria [22,23].

The following variables were analyzed: the lower esophageal sphincter (LES) pressure, wave amplitudes, and contraction abnormalities [22].

LES pressure was measured at mid-expiration and it was expressed as an average over 4 different records. Values < 10 mmHg were considered as an abnormally low LES pressure. Wave amplitudes were calculated from the mean intraesophageal baseline pressure to the wave peak. Wave amplitude < 30 mmHg was scored as hypotensive, and amplitudes < 10 mmHg were scored as failed [21]. Motility abnormalities were defined when wet swallowing was followed by repetitive and non-peristaltic waves. Repetitive waves were defined as waves composed of 3 or more peaks, with the third peak being at least 10 mmHg in amplitude and at least 1 s apart from the first. On the other hand, the simultaneous onset of contractions at 2 or more recording sites led to a non-peristaltic wave [22,23].

### 2.5. Thoracic CT Scan

A multi-slice thoracic CT scan (Lightspeed 16Pro, GE, Milwaukee, WI, USA) was performed in all patients. The scan was evaluated in double-blind by two radiologists to measure the widest esophageal diameter (WED), defined as the largest distance (mm) between internal esophageal mucosal limits according to the previous report [24]. Assessment of fibrosis and ground-glass opacities was not considered for this study.

### 2.6. Statistical Analysis

Statistical analysis was done using statistical package software (STATA 10 s). Only *p*-value < 0.05 were considered statistically significant.

Normality distribution tests were performed (Shapiro-Wilk and Kolmogorov-Smirnov) and a comparison between cytokines and clinical parameters were done using non-parametric tests (Mann-Whitney test or binomial test). All results are reported as median (IC 95%) if not else stated.

A comparison among different groups was performed using a non-parametric test (Kruskal-Wallis one-way analysis of variance). Post-hoc analysis was done using Dunn–Bonferroni tests for Kruskal-Wallis.

The correlations between cytokines and clinical parameters were analyzed by regression analysis. Spearman’s rank correlation coefficient was calculated according to the non-normal distribution of data.

## 3. Results

Twenty-nine patients (28 females, mean age 64.5 years, range 28–80) were enrolled in the study.

According to the pattern of cutaneous involvement, 17 patients were diagnosed with limited SSc (lSSc) and 12 patients received the diagnosis of diffuse SSc (dSSc).

Twenty healthy subjects (12 females, mean age 59.0 years, range 47–65) with gastric discomfort and normal upper GI endoscopy were also included as the control group.

### 3.1. Gastric Involvement

**Figure 1:**
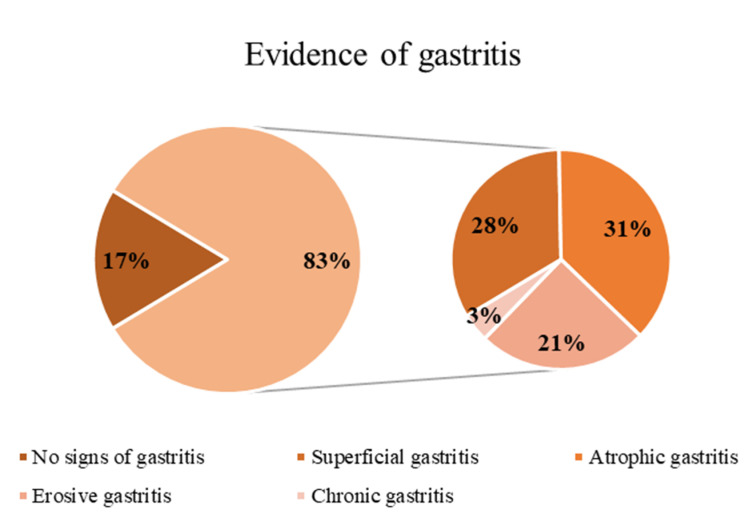
Evidence of gastritis in the whole systemic sclerosis (SSc) group.

Based on pathology examination of gastric biopsies, twenty-four patients (82.7%) received the diagnosis of gastritis: superficial in 8 patients (33.3%), atrophic in 9 subjects (37.5%), erosive in 6 patients (25.0%), and chronic gastritis in one patient (4.2%) (Figure 1, Table 1). *Helicobacter pylori* (HP) infection was detected in 5/29 patients (17.2%). No signs of gastritis were shown in gastric biopsies of healthy controls.

### 3.2. Gastric Juice Cytokines Levels

The values of cytokines in patients and healthy controls are reported in Table 2.

Significant lower levels of IL-6 (*p* = 0.001), IL-17 (*p* = 0.001), IL-22 (*p* = 0.041) and ET-1 (*p* = 0.033) were observed in patients compared with healthy controls (Table 2).

**Figure 2:**
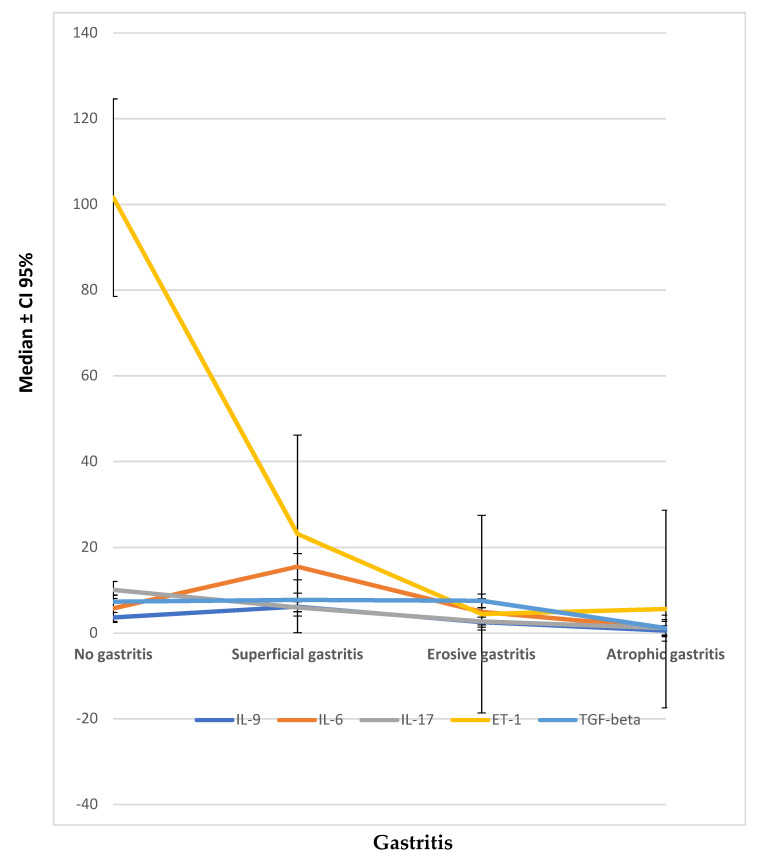
Cytokines distribution (median ± CI 95%, pg/mL) in different types of gastritis.

Patients with atrophic gastritis showed significant lower levels of many cytokines (IL-2 (*p* = 0.036), IL-9 (*p* = 0.001), IL-6 (*p* = 0.038), and TGF-β (*p* = 0.015) GM-CSF (*p* = 0.001), IL-17 (*p* = 0.001) and ET-1 (*p* = 0.001)) compared to patients without gastritis (Table 3, Figure 2).

Higher levels of IL-2 (*p* = 0.001) and ET-1 (*p* = 0.010) were observed in patients without gastritis compared with healthy controls (Table 4).

### 3.3. Esophageal Involvement

Fourteen patients (48.3%) did not complain any symptoms. The most commonly reported symptoms were odynophagia in 7 patients (24.2%), dysphagia in 11 (37.9%), regurgitation in 5 (17.2%), heartburn in 9 (31.0%) and chronic cough in 13 patients (44.8%) (Table 5).

Esophageal dilation (defined as ≥ 9 mm in the infra-aortic esophagus was found in twenty-three patients (79.3%), with a median esophageal diameter of 18.0 (14.63–20.96) mm.

LES median pressure was 10.30 (7.98–14.44) mmHg (Table 6). Peristalsis waves were synchronous in 36.4% (9.81–56.17) and hypoperistaltic in 15.4% (3.98–58.56) of cases. Median wave amplitude was 47.2 (4.61–97.57) mmHg, with a median speed of 2.5 (1.07–3.62) cm/s and a median propagation time 1.85 (1.15–10.33) s (Table 6).

Increased values of IL-2 (*p* = 0.012), IL-9 (*p* = 0.043), IL-1β (*p* = 0.045), IL-17 (*p* = 0.020), ET-1 (*p* = 0.021), and GM-CSF (*p* = 0.038) were observed in patients with esophageal impairment detected with HRM compared to patients with normal HRM (Table 7).

Overall, 18 patients out of 29 were diagnosed with any form of HRM abnormalities. Patients with HRM abnormalities were found to have significantly more frequent gastritis compared with patients without HRM abnormalities (Table 8).

## 4. Discussion

This is the first report of cytokines measurement in gastric juice of patients with systemic sclerosis.

Our results show that gastric juice concentration of the profibrotic ET-1 and the proinflammatory cytokines IL-17, IL-6, IL-1β, IL-9, and IL-2 were significantly higher in patients with esophageal dysmotility, which is known to be associated with atrophy and fibrosis of the smooth muscle layer [25,26].

ET-1 is known to be involved in initiating and maintaining the profibrotic process, by increasing collagen synthesis of many cell types, including fibroblasts and smooth muscle cells, with deposition of collagenous fibers in the interstitium of many tissues [27]. ET-1 plays a pivotal role in the chronic fibrotic process of the skin and lung of scleroderma patients [17,28].

The high ET-1 concentration we found in gastric juice of patients presenting esophageal dysmotility could be related to the collagen deposition and fibrosis of esophageal muscularis mucosa.

Th-17 cells are known to be primarily involved in inflammatory processes underlying SSc pathogenesis [29] and high levels of serum and tissue IL-17 have been associated with skin and lung fibrosis in SSc [17,30].

Hence, the high IL-17 concentrations we observed in gastric juice of scleroderma patients with esophageal dysmotility agree with these findings, so supporting the role of Th-17 cells signature not only in tissue inflammation but also in local fibrotic processes resulting in esophageal damage.

In addition, Cuiling and colleagues upheld the hypothesis that Th-17 cells act as a proinflammatory and profibrotic stimulus, but they also argue that T regulatory (T-reg) could try to stem their action [29], suggesting that an imbalance in Th17/Treg might have a more and more increasing role in SSc pathogenesis.

In our patients, we found that gastric juice levels of IL-10 and TGF-β, the two most representative T-reg cytokines, were lower in patients with esophageal dysmotility. The guess of Treg involvement in SSc pathogenesis came from the observation of a slightly lower Tregs expression in the skin of scleroderma patients compared with healthy controls and patients with local skin disease [31,32,33,34]. This led to suppose a low activity of T-regs in favor of T effector cells (Teff) in this disease [35]. Tregs are characterized by heterogeneity and plasticity, which allow them to switch to effector T cells (T-eff), particularly Th17 cells, under the stimulus of IL-6 and IL-1β, which are usually highly expressed in inflammation [31,32,33,34].

Thus, the high pro-inflammatory cytokines expression we found in patients with esophageal dysmotility could represent the optimal milieu for T-regs switching into T-eff, so explaining the lower production of IL-10 and TGF-β we found in our patients.

Finally, the significant overproduction of profibrotic factors such as IL-17, GM-CSF, and ET-1 in scleroderma patients without gastritis compared with those presenting atrophic gastritis, and the significant reduction of TGF-β, IL-6, and IL-2 in atrophic gastritis compared with patients without gastric involvement and with superficial gastritis are potentially conflicting with expectations.

However, many authors demonstrated the profibrotic role of these cytokines in scleroderma fibrotic process. Concerning IL-6, not only was it shown to induce collagen deposition through a way involving TGF-β [35], but many authors demonstrated it was also able to affect the number and frequency of Th17 and Treg cells in murine models [36].

On the other hand, Liu and colleagues analyzed Th17 and Treg expression in a cohort of new-onset SSc patients [35]. Besides an increased peripheral blood number of Th17 cells, they also found a FoxP3 (low) CD45RA (−) T cells population able to produce high levels of IL-17. Based on these observations, authors hypothesized that T cells that co-express FoxP3 and IL-17 could represent a transitional phase in the conversion process from Treg to Th17 cells [26].

Likewise, the high levels of proinflammatory cytokines, like IL-17, we found in patients with no gastritis or minimal involvement signs could correspond to the first step of the profibrotic process, starting from an attempt to the conversion of Tregs in Th-17. Lower levels of profibrotic factors as ET-1 we observed in our patients with atrophic gastritis compared to patients without gastritis are probably the consequence of a long-lasting inflammatory process leading to an end-stage fibrotic change of gastric mucosa [27].

In conclusion, esophageal dysmotility observed in SSc patients has been found to be associated with increased levels of profibrotic cytokines in gastric juice, reinforcing the role of Th17 cells in the pathogenesis of tissue fibrosis. 

The low gastric pH was shown to have no effects on cytokines production and measurement, but whether gastric cytokines measurement reflects the inflammatory pattern of esophageal and gastric scleroderma involvement should still be deepened by further observations and assessment with esophageal and gastric biopsies.

However, our study has some limitations. The study design provided, in fact, very hard exclusion criteria, mainly aiming to reduce potential drug or illness confounders, thus leading to a low sample number. In addition, gastrointestinal scleroderma involvement is primarily affecting the esophagus, being gastritis a multifactorial disease that appears in scleroderma fibrosis only in the later stages. Nevertheless, we performed cytokines measurement in gastric juice, so creating a potential detection bias.

## Figures and Tables

**Table 1:** Incidence of gastritis in the limited and diffuse SSc.

Gastritis	lcSSc (17 Patients)	dcSSc (12 Patients)	*p*-Value
	Number (%)	Number (%)	
No signs of Gastritis	3 (17.6%)	2 (16.7%)	0.453
Superficial Gastritis	4 (23.5%)	4 (33.3%)	0.109
Atrophic Gastritis	5 (20.8%)	4 (33.3%)	0.134
Erosive Gastritis	4 (23.5%)	2 (16.7%)	0.121
Chronic Gastritis	1 (4.2%)	0 (0.00%)	0.051

**Table 2:** Comparison of cytokines concentration in gastric juice (GJ) between SSc patients and healthy controls.

Cytokines in GJ	Patients Cytokines Concentration, pg/mL Median [CI 95%]	Healthy Controls Cytokines Concentration, pg/mL Median [CI 95%]	*p*-Value
IL-2	15.92 [1.48–21.75]	2.84 [2.17–3.15]	0.084
IL-9	1.37 [1.26–5.56]	3.39 [3.25–3.59]	0.279
IL-10	1.44 [0.03–2.39]	1.99 [1.78–2.31]	0.217
GM-CSF	66.38 [15.61–152.77]	1287.57 [1024.4–1304.53]	0.844
IFN-γ	36.96 [0.69–118.03]	23.47 [20.79–24.63]	0.386
TNF-α	2.45 [2.05–54.75]	1.83 [1.45–2.77]	0.460
IL-23	4.68 [2.72–53.14]	8.57 [8.41–8.78]	0.122
IL-1β	9.60 [0.88–121.70]	0.96 [0.38–2.31]	0.188
IL-6	1.36 [0.01–10.90]	5.86 [5.39–6.6]	**0.001**
IL-17	0.88 [0.34–2.25]	10.57 [8.99–11.2]	**0.001**
IL-21	14.71 [8.72–79.92]	10.04 [8.46–10.6]	0.052
IL-22	2.51 [0.23–5.60]	4.37 [3.91–6.15]	**0.041**
ET-1	10.09 [0.54–16.31]	55.92 [44.57–70.29]	**0.033**
TGF-β	7.50 [3.40–10.29]	5.35 [5.06–8.07]	0.106

**Table 3:** Comparison of cytokines concentrations in gastric juice (GJ) among different types of gastritis in SSc patients.

Cytokines in GJ	No Gastritis Cytokines Concentration. ng/mL Median [IC 95%]	Superficial Gastritis Cytokines Concentration. ng/mL Median [IC 95%]	Erosive Gastritis Cytokines Concentration. ng/mL Median [IC 95%]	Atrophic Gastritis Cytokines Concentration. ng/mL Median [IC 95%]	*p*-Value Univariate ANOVA
IL-2	7.83 [6.88–7.99] ^†^	19.30 [2.59–56.75]	7.66 [5.87–27.03]	5.07 [1.71–21.32] ^†^	0.036 ^†^
IL-9	3.32 [3.27–3.66] ^†^	6.17 [2.28–10.85] *	2.56 [1.35–10.78]	0.58 [0.24–0.83] ^†,^*	0.001 ^†^; 0.048 *
IL-10	1.65 [1.6–1.83]	2.01 [1.17–15.10]	2.24 [1.78–9.11]	1.03 [0.14–2.20]	n.s.
GM-CSF	1299.7 [611.14–1460.76] ^†,^*	451.66 [112.82–535.92] ^†^	66.38 [14.15–1374.34] *	122.30 [88.03–156.65]	0.001 ^†,^*
IFN-γ	23.07 [22.68–23.88]	79.71 [9.08–297.68]	121.23 [79.20–283.86]	62.58 [1.28–115.78]	n.s.
TNF-α	1.32 [1.05–1.52]	6.42 [6.16–57.22]	2.45 [0.84–19.02]	10.96 [7.48–74.48]	n.s.
IL-23	8.71 [5.87–11.18]	15.23 [7.84–33.48]	3.72 [2.22–32.51]	24.60 [10.55–67.21]	n.s.
IL-1β	0.65 [0.48–0.82]	21.71 [10.59–239.66] *	19.89 [5.75–73.82]	0.16 [0.08–16.79] *	0.043 *
IL-6	5.78 [3.95–7.3] ^†^	15.51 [2.44–32.47] *	4.97 [3.03–22.35]	1.17 [0.49–3.96] ^†,^*	0.038 ^†^; 0.003 *
IL-17	11.08 [10.09–11.15] ^†^	5.99 [2.73–10.77] *	2.71 [1.80–7.20]	1.17 [0.11–1.92] ^†,^*	0.001 ^†^; 0.048 *
IL-21	10.81 [10.36–11.12]	84.02 [45.64–108.25]	14.71 [6.74–97.50]	23.09 [12.68–105.29]	n.s.
IL-22	4.34 [4.06–5.09]	5.95 [2.56–8.74]	2.97 [1.05–12.02]	3.49 [0.23–7.30]	n.s.
ET-1	98.65 [91.06–102.23] ^†,^*	23.13 [3.75–51.86] ^†^	4.43 [3.75–14.40] *	5.61 [2.32–20.04]	0.005 ^†^; 0.002 *; 0.001
TGF-β	7.34 [7.04–8.27]	7.75 [7.04–8.29] ^†^	7.54 [5.73–10.38] *	1.16 [0.90–9.53] ^†,^*	0.013; 0.015 ^†^; 0.040 *

^†^: *p* < 0.05, *: *p* < 0.05.

**Table 4:** Comparison of cytokines concentration in gastric juice (GJ) between SSc patients without gastritis and healthy controls.

Cytokines in GJ	Patients without Gastritis Cytokines Concentration, pg/mL. Median [CI 95%]	Healthy Controls Cytokines Concentration, pg/mL. Median [CI 95%]	*p*-Value
IL-2	7.83 [6.88–7.99]	2.84 [2.17–3.15]	**0.001**
IL-9	3.32 [3.27–3.66] ^†^	3.39 [3.25–3.59]	0.656
IL-10	1.65 [1.6–1.83]	1.99 [1.78–2.31]	0.059
GM-CSF	1299.7 [611.14–1460.76]	1287.57 [1024.4–1304.53]	0.214
IFN-γ	23.07 [22.68–23.88]	23.47 [20.79–24.63]	0.731
TNF-α	1.32 [1.05–1.52]	1.83 [1.45–2.77]	0.021
IL-23	8.71 [5.87–11.18]	8.57 [8.41–8.78]	0.723
IL-1β	0.65 [0.48–0.82]	0.96 [0.38–2.31]	0.543
IL-6	5.78 [3.95–7.3]	5.86 [5.39–6.6]	0.463
IL-17	11.08 [10.09–11.15]	10.57 [8.99–11.2]	0.723
IL-21	10.81 [10.36–11.12]	10.04 [8.46–10.6]	0.068
IL-22	4.34 [4.06–5.09]	4.37 [3.91–6.15]	0.780
ET-1	98.65 [91.06–102.23]	55.92 [44.57–70.29]	**0.010**
TGF-β	7.34 [7.04–8.27]	5.35 [5.06–8.07]	0.453

^†^: *p* < 0.05.

**Table 5:** Symptom complaints in different SSc groups.

	Whole SSc Group (29 Patients)
Symptom Complaint	Incidence, Number (%)
No symptoms	14/29 (48.3%)
Odynophagia	7/29 (24.1%)
Dysphagia	11/29 (37.9%)
Regurgitation	5/29 (17.2%)
Heartburn	9/29 (31.0%)
Chronic cough	13/29 (44.8%)

**Table 6:** Esophageal abnormalities in SSc patients.

	Patients Group (29 Patients)
Esophageal Involvement	Median [CI 95%]
Esophageal dilation (mm)	18.0 [14.63–20.96]
Lower Esophageal Sphincter pressure (mmHg)	10.30 [7.98–14.44]
Peristalsis synchronous waves (%)	36.4 [9.81–56.17]
Hypotensive peristalsis waves (%)	15.4 [3.98–58.56]
Wave amplitude (mmHg)	47.2 [4.61–97.57]
Wave speed (cm/s)	2.5 [1.07–3.62]
Propagation time (s)	1.85 [1.15–10.33]

**Table 7:** Gastric juice cytokines concentration (pg/mL) in SSc patients based on esophageal impairment detected through High-Resolution Manometry (HRM).

	Normal HRM Cytokines Concentration, pg/mL. Median [CI 95%]	HRM Abnormalities Cytokines Concentration, pg/mL. Median [CI 95%]	*p*-Value
IL-2	7.84 [11.17–69.79]	8.95 [8.08–20.12]	**0.012**
IL-9	2.56 [0.87–7.21]	3.65 [0.71–8.86]	**0.043**
IL-10	1.73 [0.88–2.79]	1.71 [3.04–20.07]	0.150
GM-CSF	103.67 [54.06–419.34]	489.89 [122.16–1213.64]	**0.038**
IFN-γ	79.71 [39.57–100.76]	65.22 [38.22–384.27]	0.781
TNF-α	6.42 [1.6–25.28]	5.37 [14.19–76.53]	0.534
IL-23	12.51 [7.11–29.29]	9.67 [0.49–40.13]	0.868
IL-1β	19.89 [5.03–55.74]	66.51 [39.16–317.14]	**0.045**
IL-6	1.36 [1.28–10.48]	9.64 [2.07–39.9]	0.064
IL-17	2.71 [1.63–4.95]	10.09 [2.38–14.38]	**0.020**
IL-21	52.27 [24–74.5]	39.16 [0.91–109.62]	0.781
IL-22	3.49 [2.57–5.57]	5.09 [1.99–10.26]	0.359
ET-1	4.43 [0.03–32.94]	41.84 [0.11–95.73]	**0.021**
TGF-β	7.75 [3.79–8.6]	7.28 [6.71–7.73]	0.174

**Table 8:** Distribution of gastritis in patients with or without HRM abnormalities.

Type of Gastritis	Patients with Normal HRM (Number, %)	Patients with HRM Abnormalities (Number, %)	χ^2^ Test
No signs of Gastritis	3/29, 10.34%	2/29, 6.9%	<0.001
Gastritis	8/29, 27.6%	16/29, 55.17%	

## References

[1] Gu Y.S., Kong J., Cheema G.S., Keen C.L., Wick G., Gershwin M.E. (2008). The immunobiology of systemic sclerosis. Semin. Arthritis Rheum..

[2] Thoua N.M., Bunce C., Brough G., Forbes A., Emmanuel A.V., Denton C.P. (2010). Assessment of gastrointestinal symptoms in patients with systemic sclerosis in a UK tertiary referral centre. Rheumatology.

[3] Marie I., Antonietti M., Houivet E., Hachulla E., Maunoury V., Bienvenu B., Viennot S., Smail A., Dominique S., Hatron P.Y. (2014). Gastrointestinal mucosal abnormalities using videocapsule endoscopy in systemic sclerosis. Aliment. Pharm..

[4] Sjogren R.W. (1994). Gastrointestinal motility disorders in scleroderma. Arthritis Rheum..

[5] Fuschiotti P. (2016). Current perspectives on the immunopathogenesis of systemic sclerosis. Immunotargets.

[6] Chizzolini C., Boin F. (2015). The role of the acquired immune response in systemic sclerosis. Semin. Immunopathol..

[7] Leask A. (2006). Scar wars: Is TGFbeta the phantom menace in scleroderma?. Arthritis Res. Ther..

[8] Denton C.P., Abraham D.J. (2001). Transforming growth factor-β and connective tissue growth factor: Key cytokines in scleroderma pathogenesis. Curr. Opin. Rheumatol..

[9] Wang Y.Y., Wang Q., Sun X.H., Liu R.Z., Shu Y., Kanekura T., Huang J.H., Li Y.P., Wang J.C., Zhao M. (2014). DNA hypermethylation of the forkhead box protein 3 (FOXP3) promoter in CD4^+^ T cells of patients with systemic sclerosis. Br. J. Dermatol..

[10] Kataoka H., Yasuda S., Fukaya S., Oku K., Horita T., Atsumi T., Koike T. (2015). Decreased expression of Runx1 and lowered proportion of Foxp3^+^ CD25^+^ CD4^+^ regulatory T cells in systemic sclerosis. Mod. Rheumatol..

[11] Slobodin G., Rimar D. (2017). Regulatory T cells in systemic sclerosis: A comprehensive review. Clin. Rev. Allergy Immunol..

[12] Frantz C., Auffray C., Avouac J., Allanore Y. (2018). Regulatory T Cells in Systemic sclerosis. Front. Immunol..

[13] Nicola S., Fornero M., Fusaro E., Peroni C., Priora M., Rolla G., Bucca C., Brussino L. (2019). Th1- and Th17-Related Cytokines in Venous and Arterial Blood of Sclerodermic Patients with and without Digital Ulcers. Biomed. Res. Int..

[14] Manetti M., Neumann E., Müller A., Schmeiser T., Saar P., Milia A.F., Endlicher E., Roeb E., Messerini L., Matucci-Cerinic M. (2008). Endothelial/lymphocyte activation leads to rominent CD4^+^ T cell infiltration in the gastric mucosa of patients with systemic sclerosis. Arthritis Rheum..

[15] Manetti M., Neumann E., Milia A.F., Tarner I.H., Bechi P., Matucci-Cerinic M., Ibba-Manneschi L., Müller-Ladner U. (2007). Severe fibrosis and increased expression of fibrogenic cytokines in the gastric wall of systemic sclerosis patients. Arthritis Rheum..

[16] Yang X., Yang J., Xing X., Wan L., Li M. (2014). Increased frequency of Th17 cells in systemic sclerosis is related to disease activity and collagen overproduction. Arthritis Res..

[17] Rolla G., Fusaro E., Nicola S., Bucca C., Peroni C., Parisi S., Cassinis M.C., Ferraris A., Angelino F., Heffler E.L. (2016). Th-17 cytokines and interstitial lung involvement in systemic sclerosis. J. Breath Res..

[18] American College of Rheumatology (2013). 2013 Classification Criteria for Systemic Sclerosis. Arthritis Rheum..

[19] Tabeefar H., Taghi Beigmohammadi M., Reza Javadi M., Abdollahi M., Mahmoodpoor A., Ahmadi A., Honarmand H., Najafi A., Mojtahedzadeh M. (2012). Effects of Pantoprazole on Systemic and Gastric Pro- and Anti-inflammatory Cytokines in Critically Ill Patients. Iran. J. Pharm. Res. IJPR.

[20] Gannot G., Tangrea M.A., Richardson A.M., Flaig M.J., Hewitt S.M., Marcus E.M., Emmert-Buck M.R., Chuaqui R.F. (2007). Layered expression scanning: Multiplex molecular analysis of diverse life science platforms. Clin. Chim. Acta..

[21] Bredenoord A.J., Fox M., Kahrilas P.J., Pandolfino J.E., Schwizer W., Smout A.J.P.M., International High Resolution Manometry Working Group (2012). International High Resolution Manometry Working Group. Chicago classification criteria of esophageal motility disorders defined in high resolution esophageal pressure topography. Neurogastroenterol. Motil..

[22] Bassotti G., Battaglia E., Debernardi V., Germani U., Quiriconi F., Dughera L., Buonafede G., Puiatti P., Mioli P.R., Emanuelli G. (1997). Esophageal dysfunction in scleroderma: Relationship with disease subsets. Arthritis Rheum..

[23] Kahrilas P.J., Dodds W.J., Hogan W.J. (1988). Effect of peristaltic dysfunction on esophageal volume clearance. Gastroenterology.

[24] Pandey A.K., Wilcox P., Mayo J.R., Moss R., Ellis J., Brown J., Kavishwar A., Leipsic J. (2011). Oesophageal dilatation on high-resolution CT chest in systemic sclerosis: What does it signify?. J. Med. Imaging Radiat. Oncol..

[25] Rodríguez-Pascual F., Busnadiego O., González-Santamaría J. (2014). The profibrotic role of endothelin-1: Is the door still open for the treatment of fibrotic diseases?. Life Sci..

[26] Liu X., Gao N., Li M., Xu D., Hou Y., Wang Q., Zhang G., Sun Q., Zhang H., Zeng X. (2013). Elevated levels of CD4^+^CD25^+^FoxP3^+^ T cells in systemic sclerosis patients contribute to the secretion of IL-17 and immunosuppression dysfunction. PLoS ONE.

[27] Manetti M., Milia A.F., Benelli G., Messerini M., Matucci-Cerinic M., Ibba-Manneschi L. (2010). The gastric wall in systemic sclerosis patients: A morphological study. Ital. J. Anat. Embryol..

[28] Roberts C.G.P., Hummers L.K., Ravich W.J., Wigley F.M., Hutchins G.M. (2006). A case-control study of the pathology of oesophageal disease in systemic sclerosis (scleroderma). Gut.

[29] Mo C., Zeng Z., Deng Q., Ding Y., Xiao R. (2018). Imbalance between T helper 17 and regulatory T cell subsets plays a significant role in the pathogenesis of systemic sclerosis. Biomed. Pharmacother..

[30] Bălănescu P., Bălănescu E., Bălănescu A. (2017). IL-17 and Th17 cells in systemic sclerosis: A comprehensive review. Rom. J. Intern. Med..

[31] Zhou L., Littman D.R. (2009). Transcriptional regulatory networks in Th17 cell differentiation. Curr. Opin. Immunol..

[32] Klein S., Kretz C.C., Ruland V., Stumpf C., Haust M., Hartschuh W., Hartmann M., Enk A., Suri-Payer E., Oberle N. (2011). Reduction of regulatory T cells in skin lesions but not in peripheral blood of patients with systemic scleroderma. Ann. Rheum. Dis..

[33] Krasimirova E., Velikova T., Ivanova-Todorova E., Tumangelova-Yuzeir K., Kalinova D., Boyadzhieva V., Stoilov N., Yoneva T., Rashkov R., Kyurkchiev D. (2017). Treg/Th17 cell balance and phytohaemagglutinin activation of T lymphocytes in peripheral blood of systemic sclerosis patients. World J. Exp. Med..

[34] He Z., Wang F., Zhang J., Sen S., Pang Q., Luo S., Gwack Y., Sun Z. (2017). Regulation of Th17 differentiation by IKKalpha-dependent and -independent phosphorylation of RORgammat. J. Immunol..

[35] O’Reilly S., Ciechomska M., Cant R., M van Laar J. (2014). Interleukin-6 trans-signalling drives a STAT3-dependant pathway that leads to hyperactive transforming growth factor beta signalling promoting Smad3 activation and fibrosis via Gremlin protein. J. Biol Chem..

[36] O’Reilly S., Cant R., Ciechomska M., van Laar J.M. (2013). Interleukin-6: A new therapeutic target in systemic sclerosis?. Clin. Trans. Immunol..

